# Longitudinal changes in epigenetic age acceleration prior to type 1 diabetes onset in the Diabetes Autoimmunity Study in the Young (DAISY)

**DOI:** 10.1136/bmjdrc-2025-005725

**Published:** 2026-03-10

**Authors:** Kelly Bergstrom, Harry Smith, Randi K Johnson, Lauren A Vanderlinden, Jennifer Seifert, Kirk R Hohsfield, Ivana V Yang, Sarah D Slack, Fran Dong, Katerina Kechris, Marian Rewers, Jill M Norris, Patrick M Carry

**Affiliations:** 1 Epidemiology, Colorado School of Public Health, Aurora, Colorado, USA; 2 Orthopedics, Colorado School of Public Health, Aurora, Colorado, USA; 3 Department of Biomedical Informatics, University of Colorado Anschutz Medical Campus, Aurora, Colorado, USA; 4 Division of Rheumatology, University of Colorado Anschutz Medical Campus School of Medicine, Aurora, Colorado, USA; 5 University of Colorado Denver Barbara Davis Center for Childhood Diabetes, Aurora, Colorado, USA; 6 Biostatistics and Informatics, Colorado School of Public Health, Aurora, Colorado, USA

**Keywords:** Epidemiology, Diabetes Mellitus, Type 1

## Abstract

**Background:**

Type 1 diabetes is believed to be associated with early genetic and environmental stressors. Epigenetic age acceleration (EAA) is also associated with environmental stressors and the pathogenesis of many chronic diseases. This study explored longitudinal changes in EAA among individuals at high risk for type 1 diabetes.

**Methods:**

DNA methylation was measured longitudinally in subjects from the Diabetes Autoimmunity Study in the Young cohort, 2547 children born 1993–2006 at high risk for type 1 diabetes. Data were collected before and after islet autoimmunity (IA) seroconversion, a preclinical type 1 diabetes stage. EAA was estimated from DNA methylation using an epigenetic clock appropriate for pediatric blood samples. A linear mixed model was used to test for differences in EAA between 85 type 1 diabetes cases and 85 controls, before and after IA seroconversion.

**Results:**

Change in EAA significantly differed between cases and controls (p=0.02). EAA significantly decreased in cases, from pre-IA to post-IA seroconversion by 0.367 units (95% CI −0.64 to 0.09, p=0.01), but not in controls (0.045, 95% CI 0.23 to 0.32, p=0.75).

**Conclusion:**

These results suggest that EAA occurs in children who develop type 1 diabetes prior to IA seroconversion, highlighting the potential role of early environmental stressors in disease pathogenesis.

**WHAT IS ALREADY KNOWN ON THIS TOPIC**

- Epigenetic age is a biomarker influenced by environmental stressors and is linked to various chronic diseases.

**WHAT THIS STUDY ADDS**

- Our goal was to determine whether epigenetic age is associated with the preclinical development of type 1 diabetes (T1D) in high-risk children. We found that children who developed type 1 diabetes exhibited distinct changes in epigenetic age acceleration (EAA) before and after islet autoimmunity (IA) seroconversion. The differential change in EAA between type 1 diabetes cases and controls prior to IA suggests varying exposure to early environmental stressors may be influencing both the T1D disease process and EAA.

**HOW THIS STUDY MIGHT AFFECT RESEARCH, PRACTICE, OR POLICY**

- Understanding EAA and its use as a biomarker can enhance T1D prediction and inform future research, particularly in high-risk populations.

## Introduction

 Type 1 diabetes (T1D) is a chronic autoimmune condition that targets beta cells in the pancreatic islets that produce insulin.^1 2^ Although the etiology of T1D is not fully understood, it is suspected to be associated with genetic susceptibility and environmental stressors.

Islet autoimmunity (IA) is the preclinical phase of T1D where circulating autoantibodies can be detected. A subset of individuals with IA will progress to T1D.^3^ Younger age at IA seroconversion (SV) is associated with increased risk of progression to T1D.^4^ Several environmental factors that have been associated with IA and progression to diabetes include dietary exposures, early infections, psychological stressors, puberty, and increased height velocity.^5^ Many of these are also associated with aging and development, especially when occurring at a very young age.

Epigenetics refers to modifiable changes to DNA that do not alter the sequence itself. One common epigenetic marker is DNA methylation, where methyl groups attach to CpG (cytosine-phosphate-guanine) sites in DNA, which can interfere with the binding of transcription factors, thereby impacting downstream gene expression from the methylated region. Methylation is affected by aging, environmental exposures, genetics, and many chronic diseases. DNA methylation can mediate the biological effects of environmental exposures on gene expression, serving both as a proxy for environmentally induced changes in gene regulation and as an indicator of disease pathogenesis.^3 6^ DNA methylation has been implicated in the pathogenesis of T1D. A study by Raykan *et al* in discordant monozygotic twin pairs identified 132 CpG sites that were linked with T1D. The researchers found that differences could be found after IA SV, but before a diabetes diagnosis.^7 8^ Moreover, we found differences in average methylation and change in methylation at several CpG sites and regions prior to T1D diagnosis in a case-control study in the Diabetes Autoimmunity Study in the Young (DAISY).^3^ If environmental risk factors lead to abnormal epigenetic patterns and stress the pancreatic beta cells, which in turn lead to functional decline and an autoimmune attack, then these abnormal epigenetic patterns may serve as early indicators of a cumulation of environmental risks and their negative impact on the pancreas.

One theory, the beta-cell stress hypothesis, posits factors that increase stress on the pancreatic beta cells, leaving them more vulnerable to production of antigens that promote autoimmunity.^5^ Moreover, cellular senescence, where cells cease proliferation and contribute to tissue functional decline and aging, is a significant factor in both type 1 and 2 diabetes. Senescence has been seen to cause specific modifications to methylation, and although it is independent from aging, they can both be tracked by an epigenetic footprint.^9^ Cellular senescence in pancreatic beta cells may also be associated with the intricate relationship between aging and developing T1D.^9 10^ Investigating preclinical T1D may help us to understand etiology and future disease risk factors. Epigenetics is a promising avenue for exploring the connection between environmental factors and the risk of developing diabetes. To measure these changes to DNA methylation, we use epigenetic clocks. Epigenetic clocks estimate biological age from DNA methylation at various CpG sites. Epigenetic clocks are created from training sets, which determine what tissues the clock can be used on and inform how the results are interpreted. The first pan-tissue epigenetic clock was developed by Steve Horvath in 2013. It is designed to be applicable for ages 0–100 years old and on a wide range of tissue types.^6 11^ Although they were originally developed to predict chronological age, they now also function as biomarkers of biological aging and disease risk. Methylation also changes in response to environmental stress, genetics, and health stressors such as chronic disease. As such, the estimated age, or ‘epigenetic age’, may be different from the actual, which is called ‘epigenetic age acceleration’ (EAA). A higher EAA has been associated with a higher risk of developing many chronic diseases and a higher likelihood of poor outcomes. For example, higher EAA has been linked to higher cancer incidence and mortality, lower physical and cognitive fitness, and a strong correlation has been seen between obesity in greater EAA liver tissues.^12^,^14^ EAA, which accounts for chronological age variation in cell type, is known as intrinsic EAA’ (IEAA). EAA that is adjusted for chronological age as well as immune cell composition is called ‘extrinsic EAA’ (EEAA). EEAA can be used to assess functional decline of the immune system associated with aging, whereas IEAA is independent of age-related changes in blood cell composition and is moderately preserved across cell types within the same subject.^6 15 16^

The Horvath clock has shown a correlation with all-cause mortality, even after controlling for other mortality factors such as age, socioeconomic status, alcohol, smoking, cancer history, hypertension, and cardiovascular disease. The increase in Horvath EAA in children has been associated with several putative risk factors for IA and T1D, including early psychological stressors, weight gain and overweight, and an increased height velocity.^5 6 11^

In the context of T1D research, epigenetic clocks may be valuable for identifying early biological aging that precedes clinical symptoms, which can offer insight by uncovering preclinical biological aging patterns and environmental stress effects that contribute to disease onset. This study aims to determine differences in accelerated aging between children who develop T1D and those who do not, and to evaluate the potential of age acceleration at IA SV as a predictor for the timing of T1D diagnosis. We hypothesize that there are significant differences in accelerated aging through disease progression between the T1D cases versus the controls. Our goal is to improve the assessment of T1D risk in youth and better understand the relationship between aging, epigenetics, and the onset of T1D.

## Research design and methods

**Figure 1:**
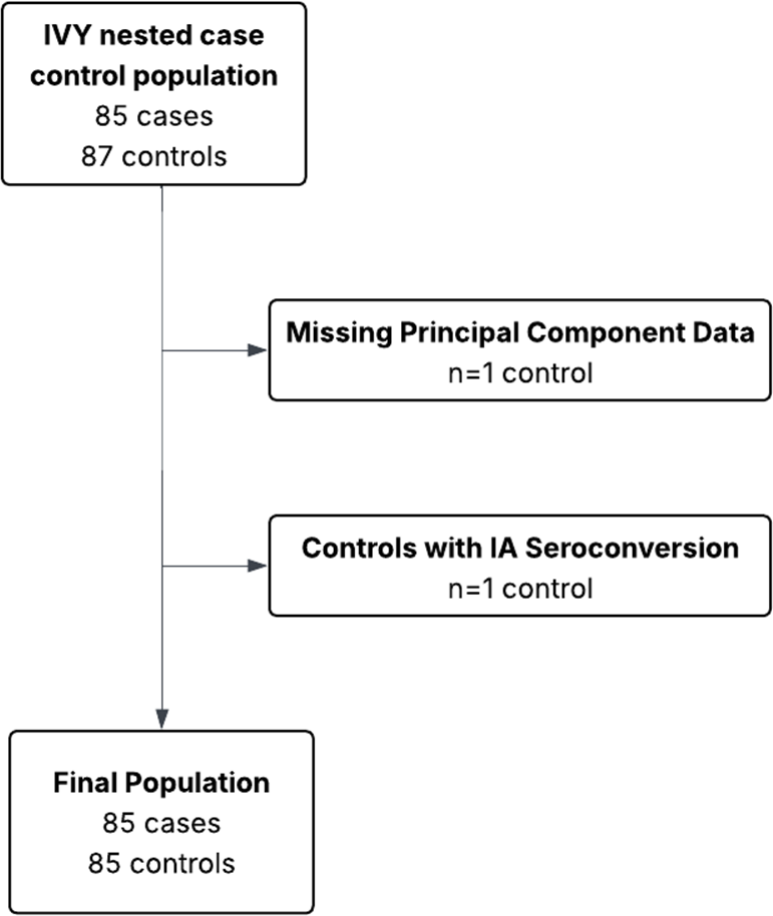
Flow chart of subject selection. Inclusion criteria involved subjects that were a member of the Investigations of Vitamins in Youth (IVY) nested-case control study. Subjects were excluded if they were missing principal component analysis data, or were controls who had had IA seroconversion. IA, islet autoimmunity.

This study used data from DAISY, a cohort of 2547 children recruited in Denver, Colorado between 1993 and 2006 at high risk for developing T1D. Participants were identified and recruited from unaffected first-degree relatives of T1D patients or by genetic screening of the cord blood of newborns at St. Joseph’s Hospital for HLA-DR genotypes, which are associated with increased risk of T1D. All newborns at this hospital were eligible for screening, except for newborns with severe congenital abnormalities or extreme prematurity, as they were more likely to have other delivery complications that decreased the ability of medical personnel to comply with the research protocol. The screening, case identification, and follow-up are detailed in earlier studies.^17 18^ Within the DAISY study, a nested case-control population was identified in January 2015, with the purpose of investigating both IA development and diabetes as endpoints. This subset was created for the Investigation of Vitamins in Youth (IVY) study. Cases of T1D were frequency matched to controls based on age of SV to IA, race/ethnicity, and sample availability for the five time points of interest over the disease course.^3^ Cases were excluded from the case-control study if they were diagnosed with T1D at birth.^3^ Cases were required to have a documented IA SV date to be included, meaning any cases that developed T1D without first developing IA were excluded (figure 1). The matching algorithm has been previously described in DAISY studies, including Johnson *et al*,^3^ with study-specific inclusion and exclusion criteria applied to align with the objectives of each individual study.

Follow-up was longitudinal and used peripheral blood samples to test for autoimmune response. Screening involved testing participants at birth, 9 months, 15 months, 24 months, and annually until autoantibodies to insulin, glutamic acid decarboxylase 65 (GAD65), IA-2, or zinc transporter 8 (ZnT8) were detected. The IA SV time point occurs when at least one autoantibody is detected at two consecutive visits. The subject’s age at the first of these two visits was defined as the age of IA SV. After IA SV, visits were more often, every 3–6 months. This continued until a physician diagnosed the subject with diabetes through either polyuria, polydipsia, and a random glucose test >11.1 mmol/L, an oral glucose test resulting in fasting plasma glucose >7.0 mmol/L, or a 2-hour glucose test resulting above 11.1 mmol/L, or to the end of follow-up in those that were not diagnosed with diabetes.^3 19^

### Study population characteristics

This analysis used the DAISY nested case-control population, which was frequency-matched for age, race/ethnicity, and number/types of visits available.^3^ Subjects were excluded for missing principal component data (n=1 control), as well as controls that IA seroconverted (n=1 control). Controls were chosen based on the absence of autoantibodies or T1D at the age when the matching case was identified with autoantibodies or T1D. The total number of participants comprises 85 T1D cases and 85 controls, of which 391 total samples were collected. Table 1 presents the sample counts and age distribution across the case and control groups at each study time point.

**Table 1:** Sample characteristics by group and visit time point

Time point	Cases		Controls	
	Samples count	Median age (Q1,Q3)	Samples count	Median age (Q1,Q3)
Early visit(t=2)	18	0.88 (0.78, 1.24)	17	0.88 (0.79, 1.24)
Pre-IA onset(t=3)	38	3.03 (0.88, 5.83)	38	2.12 (0.87, 6.32)
SV visit(t=4)	69	3.01 (1.3, 5.84)	70	2.84 (1.31, 6.01)
Pre-T1D diagnosis(t=5)	70	8.94 (5.54, 11.82)	71	8.91 (4.44, 12.01)

IA, islet autoimmunity; SV, seroconversion; T1D, type 1 diabetes.

This analysis included several key time points: an early visit (9–16 months), the pre-islet autoimmunity (IA) visit (the last sample before IA SV), the SV visit (the first detection of antibodies), and the pre-T1D visit (the last sample before diagnosis) (online supplemental appendix 1). To estimate population ancestry, a principal component analysis (PCA) was performed on DNA samples from a nested case-control study within DAISY that were genotyped using the custom-designed TEDDY-T1D (The Environmental Determinants of Diabetes in the Young-T1D) Exome array. The PCA projection analysis was performed in the reference population (controls free from T1D). The principal components 1 and 2 (PC1 and PC2) were used to adjust for ancestry; see the study by Buckner *et al*^20^ for a complete description of genotyping methods and quality control in DAISY.

The Colorado Multiple Institutional Review Board (COMIRB) approved all DAISY study protocols (COMIRB 92–080). Informed consent was obtained from the parents/legal guardians of all study participants. Assent was obtained from children 7 years or older. All research was performed in accordance with relevant guidelines and regulations.

### Epigenetic clocks

DNA methylation was quantified using the Infinium HumanMethylation450k Beadchip (450k) or the Infinium MethylationEPIC Beadchip (850k). Quantification and processing are detailed in the prior studies by Johnson *et al* and Vanderlinden *et al.*^3 21^

For this study, two clocks were considered based on their common use in the field and application to the DAISY dataset.

**Figure 2:**
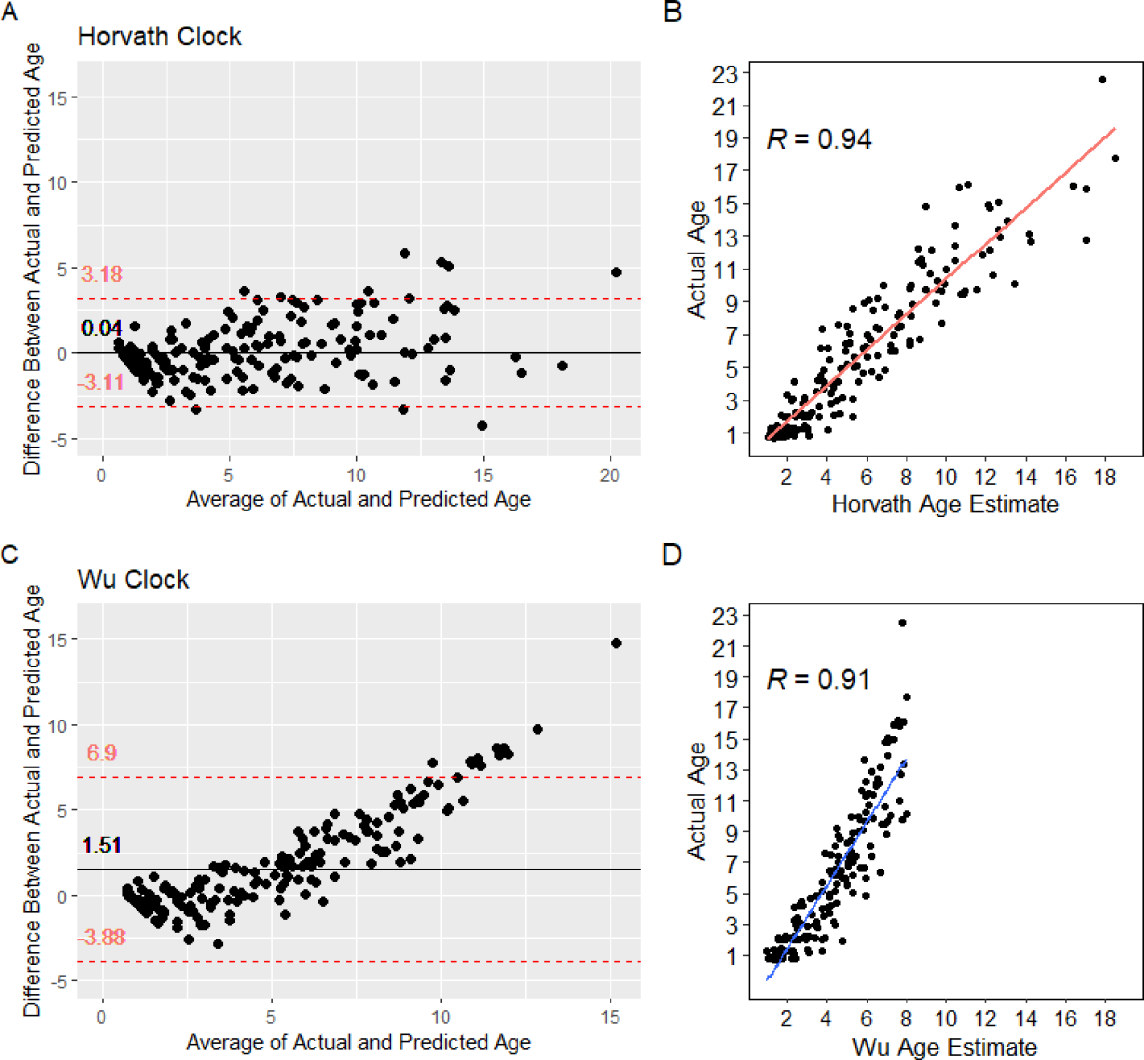
Bland-Altman plot for comparison of age and predicted age in controls for the Horvath and Wu epigenetic clocks (n=85).

The first, designed by Steve Horvath and called the ‘Horvath clock’, is widely used in the field and allows for general chronological age prediction.^6 11^ A second, known as the ‘Wu’ Clock, developed by Xiaohui Wu, was created to be specific to pediatric peripheral blood samples.^22^,^24^ R package *methylclock,* developed to compute age in years from both methylation arrays by using published epigenetic clocks, was used to calculate the predicted age and the EAA. Correlations between chronological age and actual age were compared in our control samples and a Bland-Altman plot to test for bias in measurements (figure 2).

EAA was calculated as the residuals after regressing the predicted age and actual age of a subject at the time of the sample, similar to IEAA and then directly adjusted for cell proportions within the model. A positive IEAA indicates that the epigenetic age is ahead of the chronological age.^11 15 16 25^ Two-tailed p values were considered significant if they were <0.05. A sensitivity analysis model was run using EEAA as calculated by the methylclock package. However, as we had previously derived our own cell proportion estimates for the DAISY population, we selected the IEAA model approach for consistency. The EEAA outcome, unadjusted for cell proportions, results were qualitatively similar and are provided in the Appendix for comparison (online supplemental appendix 5–7).

### Statistical analysis: linear mixed model comparing EAA before and after IA SV

IEAA was compared over time between frequency-matched cases and controls to assess whether there are significant differences in IEAA over time between children who develop T1D and those who do not.

Time points were categorized into two groups: before IA SV (early and pre-IA visits) and after IA SV but before T1D diagnosis (SV and pre-T1D visits). This was done to specifically examine the relationship with IA SV. Additionally, a model was run without collapsing the time points, and no significant change in age acceleration was found between the non-collapsed time points, supporting the use of the model with two time points (online supplemental appendix 3).

A linear mixed model was fit to account for repeated measures in each participant, allowing us to address the correlation within the same individual subjects. An unstructured covariance structure was used for flexibility of the time points, since they were not spaced evenly across subjects and were determined by clinical markers and study visit times. An interaction term between the disease progression times and T1D case/control status was included to test whether longitudinal changes in IEAA differed between cases and controls.

The model was adjusted for sex, HLA-DR3/4 genotype, the first two PCs representing genetic ancestry, cell proportions (B cell, CD4 T, CD8 T, monocytes, natural killer cells), and the type of array used (either 450k or 850k) for methylation quantification. PC1 and PC2 represent genetic ancestry and covary with family history of diabetes and race/ethnicity, which were not adjusted for in this model. Cell proportions (CD4+T cells, CD8+T cells, B cells, NK cells, monocytes, and granulocytes) were estimated using the minfi (V.1.12.0) package^26^ implementation of the Houseman method. Cell proportions were adjusted for in all models. SAS V.9.04 (SAS Institute, Cary, North Carolina, USA) and R V.4.1.0 were used for data analyses (http://www.r-project.org).

### Data and resource availability

The datasets generated during and/or analyzed during the current study are available from the corresponding author on reasonable request.

## Results

Table 2 provides descriptive data on the 170 subjects included in the T1D case versus control analysis. T1D cases were more likely to have the high-risk HLA-DR3/4 genotype (49% vs 19%, p value<0.001). Genetic ancestry variables, PC1 and PC2, significantly differed between the T1D cases and controls. The average age of SV in cases was 4.7 years old, and the controls had an average matched age of 3.8 years old at time point 4. Age at this time point was not significantly different between cases and controls (p=0 .074).

**Table 2:** Patient characteristics for case-control analysis (n=170)

Characteristic	Overall N=170*	T1D case N=85*	T1D control N=85*	P value†
Age at IA seroconversion‡	4.4 (3.6)	4.7 (3.7)	3.8 (3.3)	0.074
Sex				0.17
Male	93 (55%)	42 (49%)	51 (60%)	
Non-Hispanic white				0.82
Yes	149 (88%)	75 (88%)	74 (87%)	
First degree relative				0.084
Yes	103 (61%)	57 (67%)	46 (54%)	
HLA-DR3/4 high risk genotype				<0.001
Yes	58 (34%)	42 (49%)	16 (19%)	
Number of visits				0.96
1	41 (24%)	20 (24%)	21 (25%)	
2	59 (35%)	30 (35%)	29 (34%)	
3	48 (28%)	25 (29%)	23 (27%)	
4	22 (13%)	10 (12%)	12 (14%)	

* Mean (SD); n (%).

† Pearson’s χ2 test; Wilcoxon rank sum test.

‡ T=3 matched time point for control group.

IA, islet autoimmunity; T1D, type 1 diabetes.

To determine which epigenetic clocks would be used in the linear model, the control sample’s actual and predicted ages were compared with a Bland-Altman plot. The Wu clock displayed a systematic bias, as the average difference between measurements was 1.51, compared with 0.04 from the Horvath clock measurements. The Wu clock also showed a lower correlation coefficient (0.91) compared with the Horvath clock (0.94) (figure 2). Given the lower performance of the Wu clock on both metrics, only the Horvath clock was considered for the remainder of the analyses.

To evaluate whether IEAA differs during the preclinical phase of T1D, we analyzed longitudinal IEAA estimates in children who did or did not progress to the disease.

**Figure 3:**
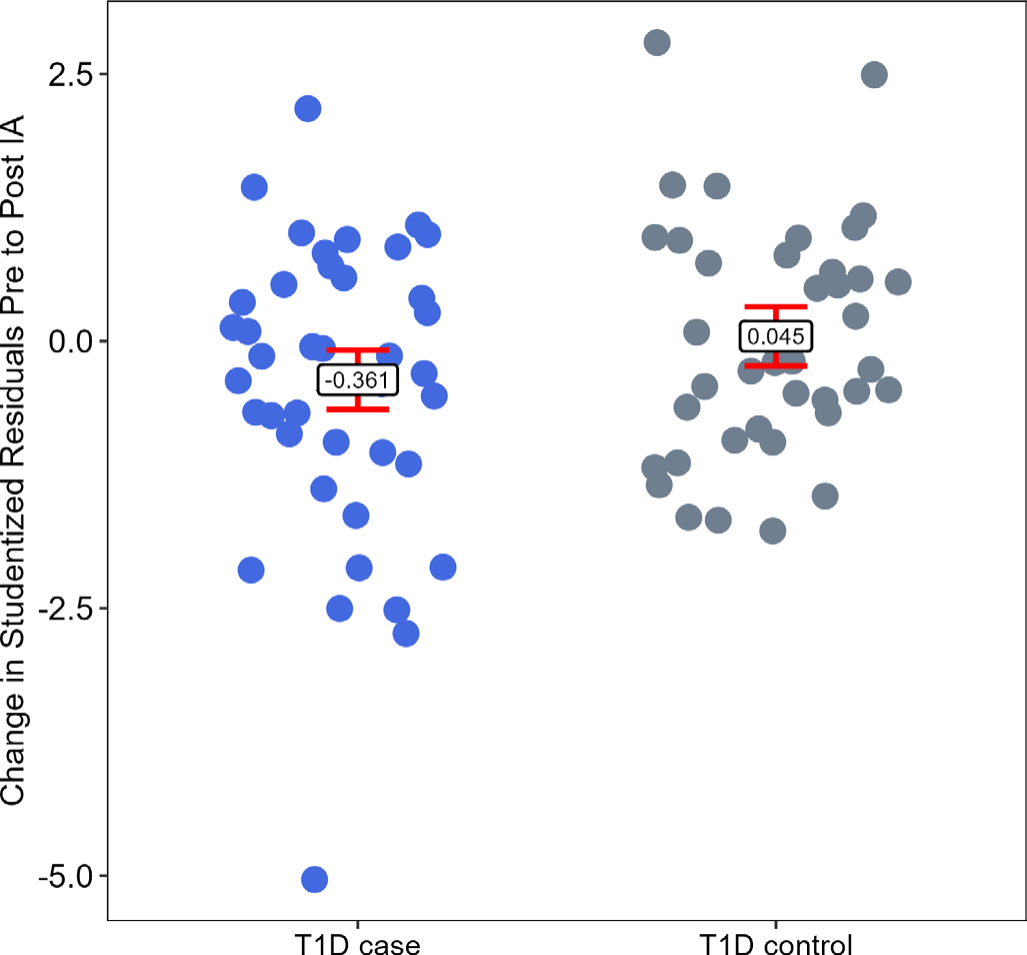
Difference in average residual before and after IA seroconversion. Points represent the subject level difference in residual. Bars represent the average change in cases and controls (error bars representing the 95% CI). IA, islet autoimmunity; T1D, type 1 diabetes.

A linear mixed model was used to compare IEAA at clinical time points before and after IA SV and to assess whether changes in IEAA over time differed between the two groups (figure 3). The Horvath clock IEAA found a significant difference in the relationship over time between T1D cases and controls, with the time point exhibiting a differential effect in the cases compared with the controls (5.135, p=0.02) (table 3).

**Table 3:** Results from linear mixed model estimating the effects of covariates on Horvath clock age acceleration

Effect	F value	P value
Averaged age at visit	13.766	<0.001**
Sex	1.571	0.21
HLA-DR3/4 high risk genotype	3.133	0.08
Principal component 1	0.421	0.52
Principal component 2	1.213	0.27
B cells	19.775	<0.001**
CD4T cells	5.467	0.02*
CD8T cells	0.178	0.67
Monocyte cells	0.085	0.77
NK cells	0.665	0.42
Array type	1.080	0.3
Disease stage	2.151	0.14
T1D group	0.476	0.49
T1D group * disease stage interaction	5.135	0.02*

T1D, type 1 diabetes.

The difference in average residual before and after IA SV was calculated for each subject (figure 3). Cases showed a significant decrease in EAA from pre-IA to post-IA SV, with an average decrease of 0.361 (95% CI –0.64 to –0.08, p=0.01). Controls exhibited a non-significant increase of 0.045 (95% CI –0.23 to 0.32, p=0.75) (figure 3, table 4).

**Table 4:** Change in adjusted age acceleration by study group and time point

Comparison	Estimate	SE	T value	P Value	Lower CI	Upper CI
Case slope: pre-IA - post-IA	−0.361	0.140	−2.573	0.011	−0.639	−0.084
Control slope: pre-IA - post-IA	0.045	0.140	0.323	0.747	−0.231	0.322
Difference: case slope versus control slope	−0.407	0.179	−2.266	0.025	−0.761	−0.052

IA, islet autoimmunity.

## Discussion

Using the Horvath epigenetic age clock, we found longitudinal differences in IEAA between T1D cases and controls, where cases displayed a different change in IEAA from before to after IA SV in T1D cases compared with frequency-matched controls. The results support that IEAA differences between T1D cases and controls exist early in the disease process, prior to IA SV.

EAA relies on predicting a person’s chronological age from DNA methylation. This is measured with ‘epigenetic clocks’, algorithms trained to find predictable epigenetic patterns that occur over time.^6^ Epigenetic clocks use the DNA methylation data collected to predict the age of the sample, also known as the epigenetic age. When the epigenetic age does not align with a person’s chronological age, an ‘accelerated’ epigenetic age indicates that there are other factors besides age influencing methylation.

Periods of rapid growth in childhood and psychological stressors have been seen to increase insulin resistance, putting higher demand on beta cells and leaving them more vulnerable to autoimmune attacks.^5^ These same stressors have also been linked to higher EAA. The differences observed in early EAA within this study may reflect the impact of environmental stressors, which are also increasing beta-cell stress and risk of developing T1D.^5^

Discrepancies between predicted and chronological age are linked to various health outcomes and disease prognosis. Prior studies have linked EAA to developmental milestones like earlier menarche age, faster weight increase, and increased height, as well as stress indicators such as cortisol and exposure to violence.^6 23 27 28^

EAA can be measured in peripheral blood samples, and with existing clocks, provide a simple and non-invasive biomarker for insight into early stages of T1D. In this study, elevated EAA before IA SV supports its potential as an indicator of increased T1D environmental risk.

The genes associated with the CpG sites used for Horvath’s clock were found to have a significant association with cell death and survival, cellular growth and proliferation, cancer, endocrine system disorder, and hereditary disorders.^11^ However, the CpG sites used for EAA calculation are not necessarily involved as a biological mechanism of T1D, as they were not selected to be predictors of T1D risk. Understanding how and why age-related methylation sites relate to T1D could provide insight on how environmental risk factors are influencing development and can help inform future research direction.^11 22 23^

Each epigenetic clock possesses strengths and weaknesses and can be trained for different ages, outcomes, and cell types. The two clocks considered, the Horvath and the Wu, were both validated in peripheral blood samples and trained on pediatric datasets. There are only three CpG sites in common between the two clocks (353 CpG sites in the Horvath clock and 111 sites in the Wu clock).

When the control samples were analyzed with a Bland-Altman Plot, the Wu clock showed a larger difference in predicted versus chronological age compared with the Horvath clock (figure 2). While the Wu clock has been shown to closely predict chronological age in pediatric populations, particularly when compared with the Horvath clock in twin studies, this strength may not translate to the goals outlined in this study.^22 23^ The difference may lie in what each clock captures. The Wu clock relies on CpG sites primarily located in genes tied to biological development and aging, which may explain its high correlation with chronological age.^22 23^ In contrast, the Horvath clock includes a broader set of CpG sites, which may better reflect environmental influences on the epigenome. This could account for its stronger performance in this study, where detecting environmentally driven variation was likely more relevant than strictly aligning with chronological age.

The Horvath clock displayed a decrease down to the control level in average IEAA after IA SV in the case group. Other early exposures, like birth weight, are not associated with Horvath EAA at birth but are positively associated in childhood and negatively associated in adolescence. Children who exhibit faster ‘aging’ when they are younger have been shown to experience a deceleration as they get older.^6 27^ Other stressful conditions have shown reversals of epigenetic age after the stressful event concludes. For instance, epigenetic age in expecting mothers increased during pregnancy and then decreased 6 weeks post partum. These results were also seen in mouse models.^29^ A severe COVID-19 infection has a similar effect on EAA, with a significant decrease after discharge from ICU.^29^ The pattern seen in this study may be similar, with cases that have a higher EAA when they are younger reverting back to the mean as they age. It is possible that the Horvath IEAA in this study is initially increased in response to the factors that are also increasing risk of IA SV, but EAA is not affected by the presence of islet autoantibodies, and thus the EAA can begin to revert to a normal level.

### Limitations

The sample size is small, making it difficult to discern significant differences in effects, especially at the epigenetic scale. The sample has low racial and ethnic diversity, with 88% non-Hispanic white subjects. Further specificity of race and ethnicity was not possible because of the small number of subjects that did not identify as non-Hispanic white. However, this racial and ethnic makeup does reflect populations that are at a higher risk for T1D. Additionally, since the children were recruited into DAISY based on being at increased risk for T1D, the results of this study are generalizable to children at high risk for T1D.

Epigenetic aging is only a biomarker and is not reflective of the complex aging and methylation process. Epigenetic clocks exist for different data sets and purposes, and it is possible that other clocks better reflect processes associated with T1D risk or have not yet been developed into an epigenetic clock algorithm. EAA is a potential indicator of risk, but it is not a risk factor itself. Development in early life also complicates the use of EAA in pediatric populations. Researchers have theorized that newborns and young children at an accelerated epigenetic age may correlate with faster maturation.^28^ However, a positive EAA is also associated with poorer disease outcomes and higher exposure to unfavorable exposures in adults.^23^ This needs to be considered when interpreting EAA in pediatric populations. Further, as this study cannot establish causality, it remains possible that subclinical IA leads to changes in blood cell composition that are captured by EAA. However, if subclinical IA occurs prior to EAA, the ability to detect EAA earlier than IA SV would still render it a valuable indicator.

### Strengths

The longitudinal nature of the DAISY study is invaluable for tracking age acceleration changes over time and disease stages, especially for a disease like T1D, that is most commonly developed in childhood. Children in the DAISY study were closely monitored with many study visits, providing detailed preclinical data. There were multiple time points before and after IA SV, including between IA SV and T1D diagnosis, features unique to the DAISY study.

EAA has not been used in many longitudinal studies, or in studies of T1D, making this a very novel design. The longitudinal data allow us to see when EAA changes are occurring, especially before IA, as this may have more impact on T1D development and set groundwork for future studies.

## Conclusions

This study investigated T1D preclinical trends in EAA, a concept where methylation patterns can be correlated with disease risk and prognosis. Results showed that differences in EAA may precede islet autoantibody detection. This suggests that early environmental factors that influence epigenetic age also impact disease risk, and this level of difference can be measured with epigenetic clocks. Understanding these biomarkers can enhance T1D prediction and inform future research, particularly in high-risk populations.

## Supplementary material

10.1136/bmjdrc-2025-005725 online supplemental file 1

## Data Availability

Data are available upon reasonable request.
