# Supplementary material for: Longitudinal changes in epigenetic age acceleration prior to type 1 diabetes onset in the Diabetes Autoimmunity Study in the Young (DAISY)
Source: BMJ Open Diabetes Res Care. 2026 Mar 10;14(2):e005725. doi: 10.1136/bmjdrc-2025-005725 (PMC12983902; doi:10.1136/bmjdrc-2025-005725)
Supplement: online supplemental file 1 [file bmjdrc-14-2-s001.docx]

# Supplemental Material:

### Appendix 1: Study Design and Disease Progression


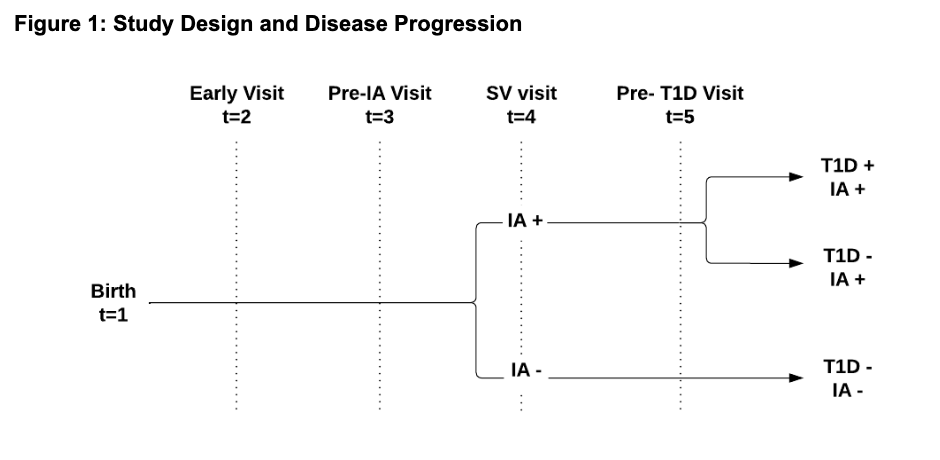


*Appendix 1: Time points as defined in this study 2=between 9 and16 months old, 3= last visit before IA seroconversion, 4= first visit where IA seroconversion is identified, 5= last visit before type 1 diabetes diagnosis.*

###

###

###

###

###

### Appendix 2: Adjusted Age Acceleration by Group

|  | | | | |
| --- | --- | --- | --- | --- |
| Group | **Pre IA** | | **Post IA** | |
|  | Age Acceleration (CI) | P Value | Age Acceleration (CI) | P Value |
| Control | 1.884(0.85,2.92) | <0.001** | 1.929(0.92,2.94) | <0.001** |
| Case | 2.189(1.21,3.17) | <0.001** | 1.828(0.83,2.83) | <0.001** |

### Appendix 3: Subject Age Distribution by Group and Time Point

###
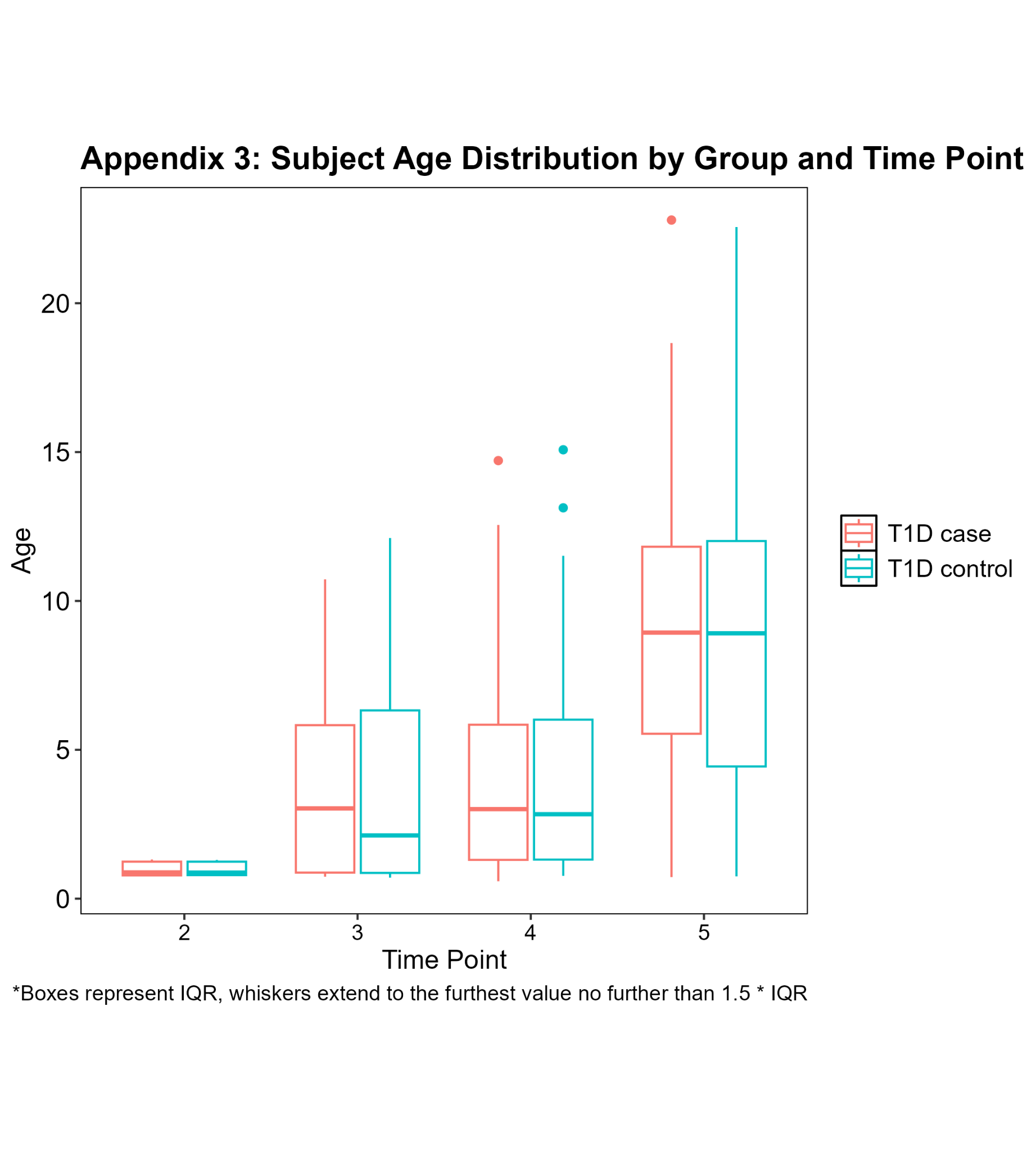


*Appendix 3: Age distribution of subjects at each study time point. Red= case group, Blue = control group. 2= between 9 and16 months old, 3= last visit before IA seroconversion, 4= first visit where IA seroconversion is identified, 5= last visit before type 1 diabetes diagnosis.*

### Appendix 4: Chronological age vs Age Acceleration in Cases and Controls


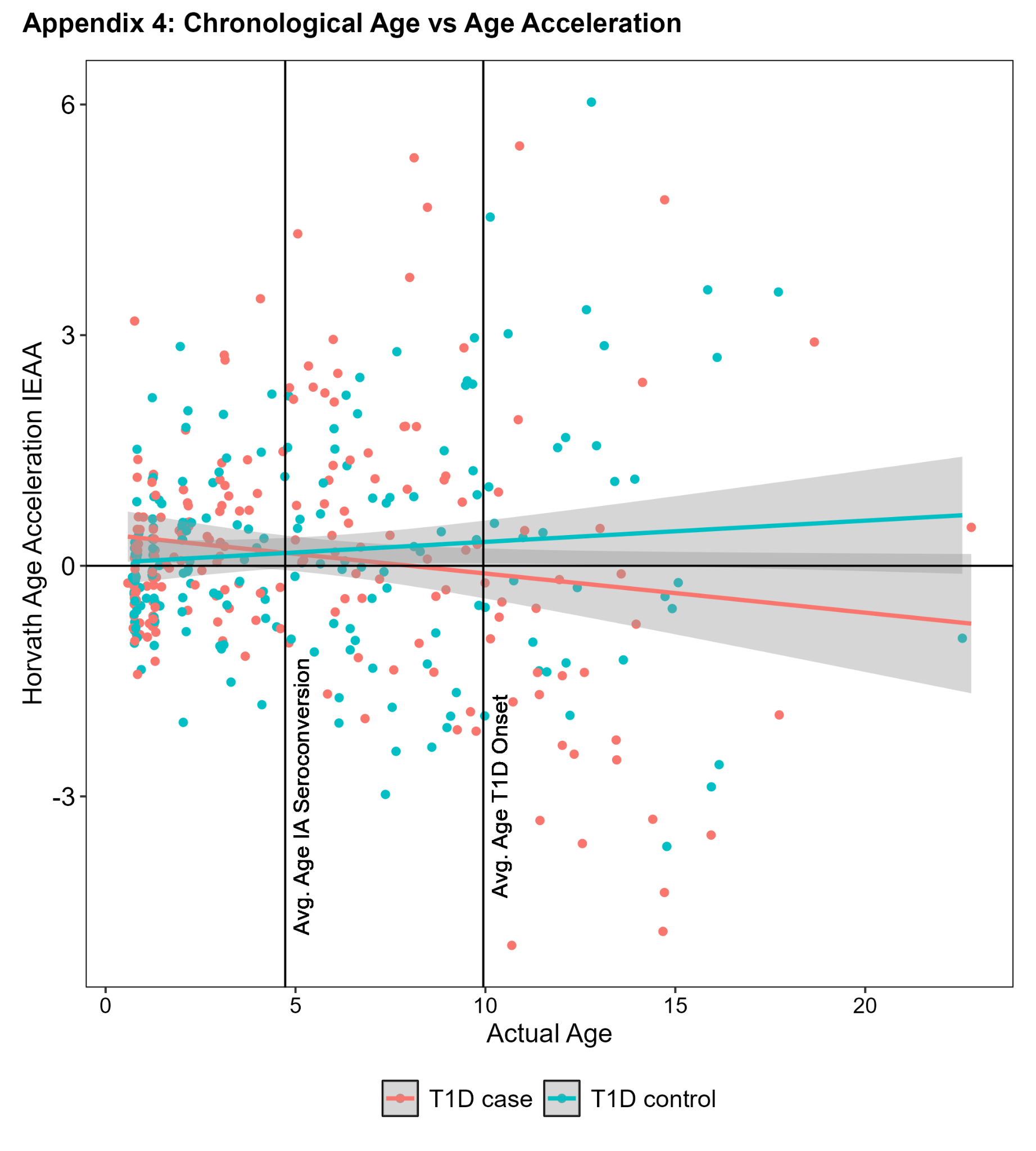


*Appendix 4: Chronological Age compared to the IEAA calculated by the Horvath clock, and a simple linear model comparing them for cases and controls. Gray area represents 95% confidence internals. Red= case group, Blue = control group*

### Appendix 5: Type 3 Fixed Effects-Pre and Post IA: EEAA from Methylclock Package

| Effect | F Value | P Value |
| --- | --- | --- |
| Age | 0.000 | 0.99 |
| Sex | 0.316 | 0.57 |
| DR 3/4 High Risk Genotype | 0.948 | 0.33 |
| Principal Component 1 | 0.101 | 0.75 |
| Principal Component 2 | 0.237 | 0.63 |
| Array Type | 2.172 | 0.14 |
| Disease Stage | 1.696 | 0.19 |
| T1D Group | 0.090 | 0.76 |
| T1D Group * Disease Stage Interaction | 4.403 | 0.04* |

| Appendix 6: Change in Age Acceleration by Group and Time Point: EEAA from Methylclock Package | | | | | | |
| --- | --- | --- | --- | --- | --- | --- |
| Comparison | Estimate | Standard Error | T Value | Pr > \|t\| | Lower CI | Upper CI |
| Case Slope: Pre-IA - Post-IA | −0.326 | 0.140 | −2.328 | 0.021 | −0.603 | −0.050 |
| Control Slope: Pre-IA - Post-IA | 0.051 | 0.137 | 0.370 | 0.712 | −0.220 | 0.322 |
| Difference: Case Slope vs Control Slope | −0.377 | 0.180 | −2.098 | 0.037 | −0.732 | −0.022 |


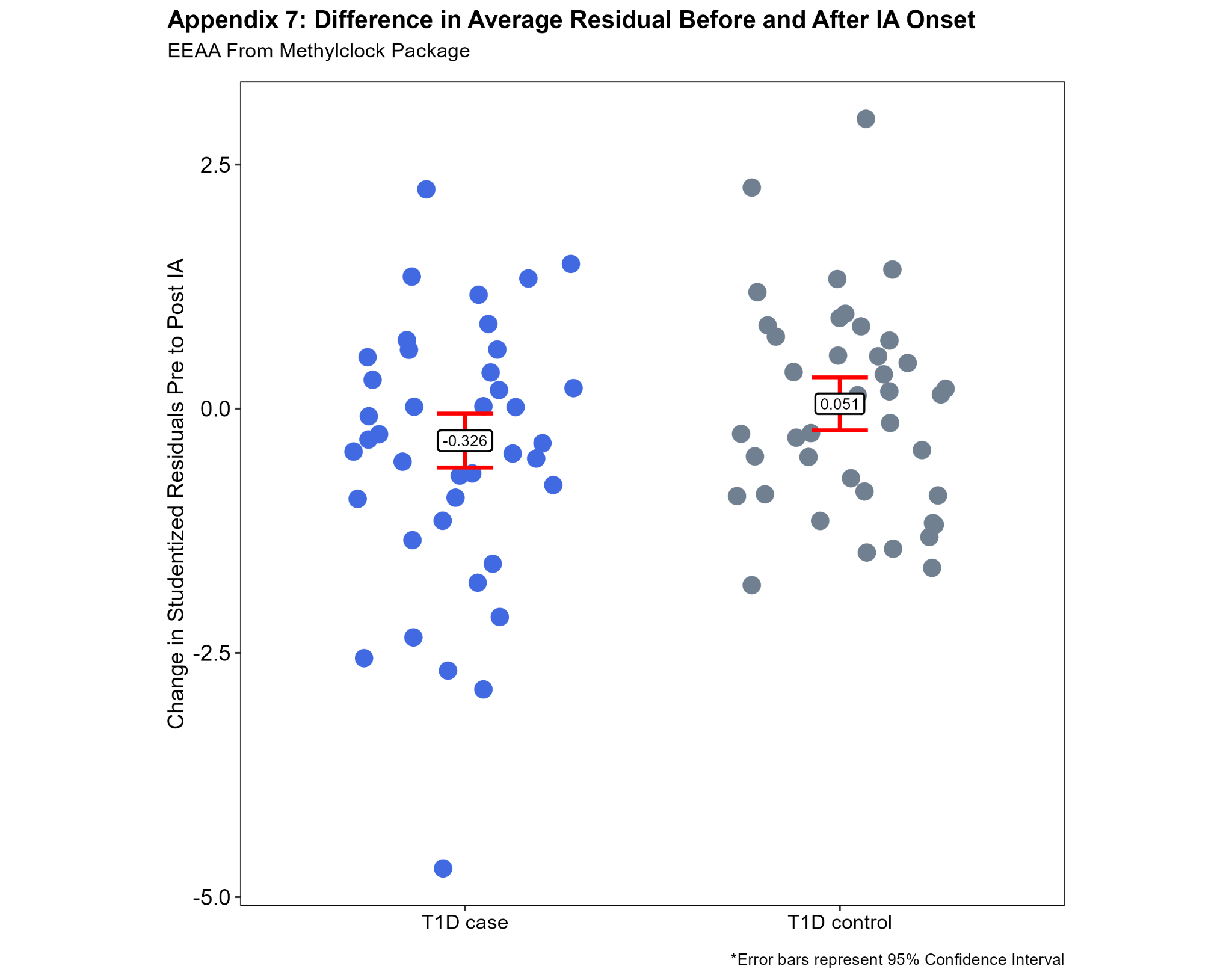


### Appendix 7: Sensitivity analysis, the difference in average residual before and after IA onset, using EEAA as the outcome. Points represent the subject level difference in residual. Bars represent the average change in cases and controls (Error bars representing the 95% Confidence Interval). Blue circles = case group, gray circles= control group
